# Analyzing the effect of the COVID-19 vaccine on Parkinson’s disease symptoms

**DOI:** 10.3389/fimmu.2023.1158364

**Published:** 2023-06-05

**Authors:** Revati Sabat, Orrin L. Dayton, Amit Agarwal, Vinata Vedam-Mai

**Affiliations:** ^1^ Eastside High School, Gainesville, FL, United States; ^2^ Department of Radiology, University of Florida, Gainesville, FL, United States; ^3^ Department of Radiology, Mayo Clinic, Jacksonville, FL, United States; ^4^ Department of Neurology, University of Florida, Gainesville, FL, United States

**Keywords:** Parkinson’s disease, COVID-19, vaccine, movement, neurodegenerative

## Abstract

**Background:**

Parkinson’s Disease (PD) is one of the most common neurodegenerative diseases. PD has recently received more attention by researchers in the midst of the COVID-19 pandemic.

**Objective:**

Yet to be researched is the effect of the COVID-19 vaccines on PD patients. Several PD patients are still hesitant to the vaccine due to this unaddressed fear. The purpose of this study is to address this gap.

**Methods:**

Surveys were administered to PD patients 50 years and older at UF Fixel Institute who received at least one dose of the COVID-19 vaccine. Survey questions included patients’ severity of PD symptoms before and after the vaccine and extent of worsening PD symptoms post-vaccination. After three weeks of collecting responses, the data was analyzed.

**Results:**

34 respondents were eligible for data consideration because they fell within the age range being studied. A total of 14 respondents out of 34 (41%, p=0. 0001) reported that their PD symptoms worsened after the COVID-19 vaccine to some extent.

**Conclusion:**

There was strong evidence of worsening of PD symptoms post COVID-19 vaccination, however it was mostly mild and limited to a couple of days. The worsening had statistically significant moderate positive correlation with vaccine hesitancy and post-vaccine general side effects. A possible causative mechanism of PD symptom worsening using existing scientific knowledge would be stress and anxiety associated with vaccine hesitancy and the extent of post-vaccine general side effects (fever, chills, pain), likely via simulating a mild systemic infection/inflammation the latter already established causes of PD symptom worsening.

## Introduction

Parkinson’s Disease (PD) is one of the most common neurodegenerative diseases—an illness involving deterioration in parts of the brain controlling movement (1). It has a relentlessly progressive course, affecting over 1% of the world population, predominantly those over the age of sixty. Currently, nearly one million PD patients are living in the United States and about sixty-thousand Americans are diagnosed with PD every year (2). The average onset of PD is 60 years and the male to female ratio is 2:1. Only 4% of patients are under 50 years. Those who receive diagnosis from ages 21-50 are categorized as early onset PD. Early onset PD is characterized by slower progression and atypical symptoms (3). Being relatively prevalent among the elderly population, PD is one of the most actively researched neurodegenerative disorders (2). PD has recently received more attention by researchers in the midst of the COVID-19 pandemic. Recent research regarding PD indicates that the COVID-19 pandemic has had both physical and psychological effects on PD patients’ symptoms. However, yet to be researched is the effect of the COVID-19 vaccines (Pfizer, Moderna, Johnson & Johnson) on PD patients. Several PD patients are still hesitant to the vaccine due to this unaddressed fear. Even the American Parkinson Association has expressed their helplessness by communicating that apart from sporadic reports of transient worsening there is no systematic research on this topic yet (4). The purpose of this study is to address this gap. Levodopa with carbidopa is the most common used drug combination for PD. Levodopa, the most effective Parkinson’s disease medication, is a natural chemical that is converted to dopamine in the brain. Carbidopa protects levodopa from early conversion to dopamine outside your brain and decreases the side effects such as nausea.

PD patients can experience a variety of motor and non-motor symptoms that can vary in intensity and progression based on the individual. Cardinal motor symptoms include tremor, rigidity, bradykinesia, and postural instability. Common non-motor symptoms include loss of smell, sleep problems, depression, fatigue, personality changes, dementia, and lightheadedness (2). Within a year of the COVID outbreak, three COVID-19 vaccines have been approved and authorized by the CDC in the United States: Pfizer-BioNTech (Pfizer), Moderna, and Johnson & Johnson’s Janssen (J&J). All of the vaccines require two doses given 3 weeks apart, except for J&J which only requires one dose. According to the CDC, a vaccine recipient is considered fully vaccinated two weeks after their last shot. The Pfizer and Moderna COVID-19 vaccines are a new type of vaccines called mRNA vaccines, which contain a short fragment of mRNA to create antibodies to the SARS-CoV2 spike protein. mRNA vaccines have never been used in humans before and unlike previous vaccines, the period of development/testing of the vaccines have been the shortest in human history, keeping a wider probability of unknown side effects (4). The J&J vaccine is an adenoviral vector vaccine which incorporates spike S protein DNA in an adenovirus 26 DNA. The vaccines are very safe and efficacious with minor side effects like headache, malaise, pain at injection site. Few cases of myocarditis and pericarditis have been reported, usually in adolescents and young adult males, within several days after mRNA COVID-19 vaccination (Pfizer-BioNTech or Moderna). Few minor neurological complications have been reported following SARS-Cov-2 vaccination, without a clear causal relationship ever being verified, including some cases of worsening of Parkinson’s disease (PD). Our study aims to further confirm the negligible effect of the COVID vaccines on PD symptoms and eventually allaying vaccine hesitancy.

## Materials and methods

We hypothesized that there would be an exacerbation of PD symptoms post vaccination, based on sporadic reports of worsening by patients after vaccination (5). Therefore, the null hypothesis (H_0_) is that the vaccine does not exacerbate PD symptoms and the original hypothesis is the alternate hypothesis. Exacerbation, in this study, was defined by the criteria used in the study of Zheng et al, “Motor exacerbations were defined as patient-reported episodes of subacute worsening of PD motor function in 1 or more domains (bradykinesia, tremor, rigidity, or PD-related postural instability/gait disturbance) that caused a decline in functional status.” Spontaneous exacerbation was defined as an exacerbation occurring in absence of known inciting factors such as infection, stress, hospitalization, discontinued medication, and stress (6). The design for this study was a patient survey and was influenced by Muhaidat et al’s study on the effect of COVID-19 vaccines on menstrual symptoms (7).

### Study population

The participants of this research were PD patients at the Fixel Institiute of Neurological Diseases of University of Florida. Participants’ PD diagnoses had been confirmed by Board Certified neurologists specializing in ‘Movement Disorders’. The questionnaire was open to all patients at the institute willing to participate, but only data of those 50 years and older and those that received at least one dose of the COVID-19 vaccine was used. Young onset PD patients were excluded since they experience a different disease course, which could cause data contamination. Moreover, they represent only 4% of PD patients. Additionally, past studies on COVID-19 and PD have mostly been conducted on similar demographics, enabling better comparison.

### Study tools

The primary instruments in this study were paper copies of a Qualtrics™ survey (**Appendix B**). This survey platform was chosen due to its credibility as an approved software by the university. Paper copies were used to better suit patient demographics. The questionnaire was developed through an iterative process involving an extensive literature search that included peer reviewed PD journals and was approved by the Institutional Review Board (**Appendix A**). The survey questions were reviewed and validated by neurologists at UF.

The questionnaire consisted of 22 questions (**Appendix B**). Several of the questions were designed to collect quantitative data through the use of a Likert scale (0-5), in which subjects were able to select where they fell on the scale. The use of this scale was adopted from Denjun’s study because it allowed patients to choose ‘0’, to suggest no worsening (8).

### Data collection procedures

Study subjects were handed paper versions of the Qualtrics™ survey. An informed consent form was given before the questionnaire (**Appendix C**). Participants were also given an email and number that they could contact if they wished to withdraw their response. After 3 weeks, the surveys were hand delivered for analysis. Once analysis was complete, the paper surveys were no longer accessible.

## Results

### Demographics, disease distribution, vaccine hesitancy, COVID-19 contraction

Over the course of the three weeks, the questionnaire (**Appendix B**) was completed by 38 patients. However, four of the responses were discarded due to incomplete surveys. This left 34 patient responses for analysis (**Appendix D**). All 34 (*N*=34) respondents were eligible for data consideration because they fell within the age range being studied (50+) and received at least one dose of a COVID-19 vaccine. Out of the 34 respondents, 62% (*n*=21) were male, 38% (*n*=13) were female, and all were white. The age ranges of the participants were 50-59 (*n*=2, 6%), 60-70 (*n*=16, 47%), and 70+ (*n*=16, 6%). Over half of the participants had PD for 0-7 years (*n*=20, 59%). The number of participants who had PD in further 7-year intervals of 8-15 years and 16-23 years were equal (*n*=7, 21% each). None had disease longer than 23 years.

Seventy-one percentage (71%) patients (*n*=24) had not contracted COVID-19 during the pandemic, while only 29% (*n*=10) had tested positive. **Table 1** also depicts where the respondents fell on a scale of 0-5 regarding vaccine hesitancy, 0 being no hesitation and 5 being extreme hesitation. 18% of the participants (*n*=6) had no hesitation to receive the vaccine while 15% (*n*=5) were extremely hesitant. As shown in **Table 1**, the largest category of respondents (*n=8*, 24%) reported a ‘1’ indicating that they had slight hesitation.

**Table 1:** Vaccine hesitancy among patients on a 6-point Likert scale.

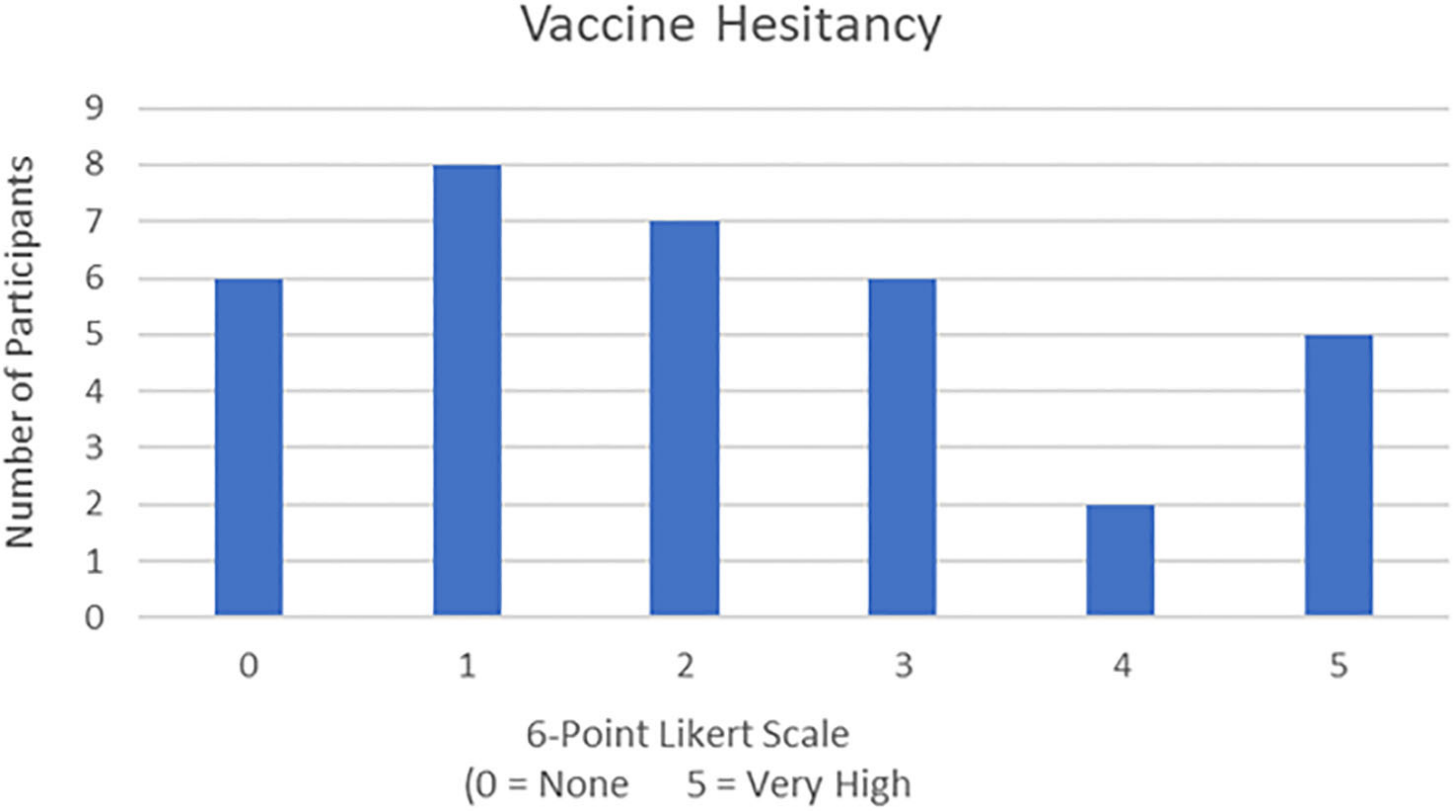

### COVID-19 vaccine information, general symptoms, PD symptoms data

Three patients (9%) had received the J&J vaccine, 10 (29%) acquired Moderna, and the remaining 21 (62%) received the Pfizer vaccine. The most common general side effects of the vaccine were drowsiness (*n*=12, 26%), fatigue (*n*=12, 26%), chills/fever (*n=*10, 21%), local swelling (*n*=7, 15%) and headaches (*n*=6, 13%). These numbers expectedly add to more than 34, since the participants often experienced more than one symptom.

**Table 2**. shows patients’ responses on their vaccine’s general side effects and severity. These general side effects reportedly lasted for less than 24 hours for 20 (59%) patients, between 24-48 hours for 9 (26%), and over 48 hrs. for 4 patients (12%). One (3%) did not experience side effects at all. Responses on baseline (before vaccination) troublesome PD symptoms and to what extent they interfered in patients’ lives can be seen in **Table 3**. At baseline, the two most common bothersome PD related symptoms were gait/balance (*n*=16) and tremors (*n*=15). Other troublesome PD symptoms included slowness (*n*=13), stiffness (*n*=11), fatigue (*n*=10), speech difficulties (*n*=9). Ten (30%) respondents expressed that these symptoms greatly (Likert score of 5) limit their activities of daily living.

**Table 2:** General side-effects of vaccine with their severity.

	Q10 Vaccine Side Effects					
	Severity Scale	Drowsiness	Chills/Fever	Headache	Swelling	Fatigue
**Side** **Effects** **Severity**	1	7	6	1	4	8
	2	1	1	2	2	1
	3	2	2	1	0	0
	4	0	0	2	0	1
	5	1	1		1	2
	0 (none)	1	0	0	0	0

**Table 3:** Troublesome PD symptoms and interference of symptoms with daily activities.

	Q14 Interference of Symptoms in Life										
	Severity Scale	0	1		2		3	4		5	Total
	Symptoms	Number of subjects									
**Troublesome PD Symptoms**	Tremors	0	2	2		2			4	5	15
	Cognitive Issues	0	0	1		1			1	1	4
	Fatigue	1	1	0		3			2	3	10
	Gait/Balance	0	1	1		4			4	6	16
	Loss of Smell	0	0	2		0			2	0	4
	Sleep Problems	0	0	0		1			0	5	6
	Slowness	1	0	1		5			1	5	13
	Speech Difficulty	0	0	2		1			2	4	9
	Stiffness	0	0	1		5			0	5	11
**Total**		1	3	5		8			7	10	34

A total of 14 respondents reported that their PD symptoms worsened after the COVID-19 vaccine to some extent (or 0-5 on the Likert Scale). Although 14 of the 34 respondents reported worsening PD symptoms, only 8 of these 14 patients felt that the worsening was severe enough to interfere with their daily activities. Specifically, 71% of patients reported that they were able to continue and 29% reported that they could not continue activities after vaccination. As seen in **Table 4**, those who reported worsening symptoms post-vaccine, the most common extent to which symptoms worsened was 2 (36%), on a scale of 0-5 (0 being not at all and 5 being to a very large extent). Also seen in **Table 4**, for those who experienced worsening symptoms, the most experienced worsening between 24-48 hours and after each dose. For those who said over 48 hours, they were asked to give an approximate point estimate for how long they experienced worsening. The 4 respondents answered 4 days, 1 week, 10 days, and 2 weeks. Furthermore, among the patients, the symptom that appeared to be worsening the most was tremors (43%). The 3 most common medications taken by the patients in this survey were Sinemet (*n*=9, 22%), Amantadine (*n*=6 15%) and Levodopa (*n*=4, 10%). Other medications that some patients took included Mirapex, Requip, Duopa, and several others. Patients were asked if they had to change their medications days leading up to or after the vaccine. Most patients (91%) did not, however, 3 patients (9%) did change their medications.

**Table 4:** Duration and extent of worsening of PD Symptoms.

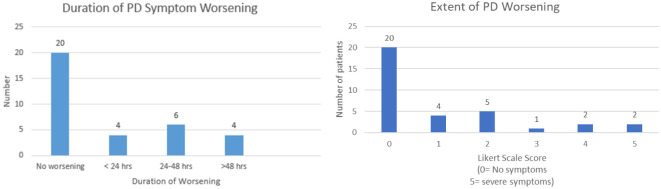

### Spontaneous baseline PD worsening data

In order to get baseline control data on spontaneous worsening without vaccine, all participants were asked whether they had experienced baseline worsening of PD symptoms in the last 12 months before the vaccine. Only 1 of the 34 patients (2.9%) reported spontaneous worsening in the past while the rest 33 (97.1%) did not recall any spontaneous PD symptom worsening.

## Statistical analyses

### Primary findings

Statistical data analysis was done using SAS™ software version 9.4M7 (SAS Institute Inc., Cary, NC). To test validity of the hypothesis by analyzing significance of the data, Fisher’s Exact Test was performed on the contingency table (**Tables 5A**, **B**) to obtain a *p* value of spontaneous PD symptom worsening before vaccination (1/34) versus after worsening (14/34). The p-value was calculated to be 0.0001. The *p* value measures the probability that an observed difference could have occurred just by random chance. In this case, p value was 0.0001, much less than the significance level (0.05), which shows high statistical significance, thus rejecting the null hypothesis and allowing acceptance of the study (alternate) hypothesis. However, as shown by the original data tables, the worsening was limited in duration as well as extent. This is displayed by the most duration being 24-48 hours (*n=* 6) and the most common response to the Likert scale being 2 (on a Likert scale of 0-5). As seen in **Table 6**, the odds ratio was 23:1. This conveys that the odds of having worsening of PD symptoms after vaccination compared to before vaccination are 23:1, which is quite high. The relative risk of patients who get the vaccine to develop worsening of their symptoms is 1.65. Relative risk- which informs the probability of a PD patient developing worsening of their PD symptoms post-vaccine is more relevant for this study.

**Table 5A:** SAS results with frequency of response.

SAS results Table of group by response			
Group	Response		
Frequency	No	Yes	Total
**pre**	33	1	34
**zpost**	20	14	34
**Total**	53	15	68

**Table 5b:** Fisher’s exact test for significance of worsening of PD symptom post- vaccination.

Fisher’s Exact Test	
**Cell (1,1) Frequency (F)**	33
**Left-sided Pr<=F**	1.0000
**Right-sided Pr>=F**	0.0001
**Table Probability (P)**	0.0001
**Two-sided Pr<=P**	0.0002

**Table 6:** Odds ratio and relative risks.

Odds Ratio and Relative Risks			
Statistic	Value	95% Confidence Limits	
**Odds Ratio**	23.1000	2.8191	189.2827
**Relative Risk (Column 1)**	1.6500	1.2380	2.1990

### Additional correlational analysis

Spearman’s Correlation Test was further performed to detect correlation among various variables to detect possible mechanisms behind the strong association between COVID-19 vaccination and worsening of PD symptoms. Spearman’s Correlation Test was preferred over supplemental tests, such as Pearson’s Correlation, because it tests monotonic association, which is to say, an association that is less restrictive than a linear association. No statistically significant correlations were found between variables such as duration of PD worsening and patient’s age with extent of PD worsening. However, there were several statistically significant, positive correlations between the variables (seen boxed in yellow). Interestingly, there were two very strong and positive correlations. The *r* value for the extent of PD worsening post-vaccine and duration of PD worsening was 0.96 (almost 1), indicating an extremely strong, positive correlation between extent of PD worsening and duration of worsening. Secondly, there was a strong, positive correlation between patients who experienced worsening after each dose and the extent to which their PD worsened (*r=*0.82). However, these correlations are features of worsening rather than causative correlations. The possible causative correlations are discussed in the discussion section below. The 95% confidence interval in **Table 7** indicates that one is 95% confident that the mean *r* value lies between the depicted range.

**Table 7:** Spearman correlation for important parameters.

Variable	r	95% C.I	P
Contracting COVID & PD worsening*	-0.2	(-0.51, -0.14)	*0.25*
Contracting & Duration of PD worsening	0.24	(-0.53, 0.11)	*0.18*
Medications & PD worsening	0.11	(-0.40, 0.20)	*0.24*
Duration of PD & PD worsening*	0.1	(-0.24, 0.43)	*0.33*
Duration of PD & Duration of Worsening	0.05	(-0.29, 0.38)	*0.76*
Age & PD Worsening*	-0.2	(-0.50, 0.15)	*0.08*
Hesitancy & Side Effect Severity**	0.28	(-0.42, 0.18)	*0.33*
Age & Duration of PD worsening	-0.23	(-0.53, 0.11)	*0.18*
Hesitancy & PD Worsening*	0.33	(-0.11, 0.42)	*0.01*
Side Effect Severity** & PD Worsening*	0.28	(-0.16, 0.49)	*0.04*
Vaccine Type & PD Worsening*	0.28	(-0.16, 0.32)	*0.02*
Vaccine Type & Duration of PD Worsening	0.32	(0.11, 0.34)	*0.003*
PD Worsening & Duration of PD Worsening	0.96	(0.93, 0.98)	*<0.001*
Worsening after each dose of PD Worsening	0.82	(0.77, 0.85)	*<0.001*

*Spearman’s Correlation value (r) is between -1 to 1. Positive value indicates positive correlation, negative value indicates negative correlation.*

*PD worsening here refers to severity of the reported worsening.

**Side effects severity refers to severity of general non-specific symptoms like fever, chills, headaches, fatigue, etc. The important relationships with higher correlation coefficients are highlighted in the yellow box.

## Discussion

Although the COVID-19 vaccine is principally an mRNA coding the spike protein, on rare occasions it can cause problems like COVID-19 disease itself via anti-idiotype antibodies, which stimulate virus-like immune pathways (9). Hence, it is important to briefly understand the effects of COVID-19 disease on PD patients. Current data suggests that PD patients who contract COVID-19 face higher mortality rates than the general population. It was found that PD patients with a diagnosis of COVID-19 and found an overall mortality rate of 18.9% versus the age adjusted baseline mortality of 3-4% in the general population (10). Such higher mortality rates have been attributed to Covid 19 induced pneumonia (11). COVID-19 has been shown to influence symptoms of PD patients. Artusi et al. also reported that COVID-19 generally worsened PD symptoms. About 37.1% of the subjects, from six studies reporting therapy information, required a higher levodopa dose while they were infected due to worsening of PD-related symptoms (10). Xu et al., surveyed 46 patients with PD and COVID-19 and found that worsening of PD symptoms during COVID-19 disease progression included bradykinesia, rigidity, and balance disturbances (12).

Pre-existing research suggests that beyond affecting physical health, the COVID-19 pandemic has additionally interfered with PD patients’ mental health and daily living. A meta-analysis of 31 studies by King’s College London researchers led to the conclusion that COVID-19 had increased symptoms such as anxiety, depression, sleep problems, loss of motivation, and stress in PD patients (13). The COVID-19 vaccine has been known to worsen, or even cause, other medical conditions. A recent study by Block et al., conducted via the gathering of electronic health data of patients from 40 health care systems, discovered that COVID-19 vaccines can cause heart complications (like myocarditis and pericarditis—swelling and inflammation in layers of the heart) in several patients, especially children, the highest incidence being among teenagers after the second vaccine dose (14). Fortunately, by calculating risk ratios to compare risk for cardiac outcomes after SARS-CoV-2 (the virus that causes COVID-19) infection to that after COVID-19 vaccination, the study concluded the overall incidence of these COVID-19 vaccine induced cardiac complications have been very rare, prompting the strong case for vaccination despite the rare, occasional side effects. These findings were confirmed through the means of a clinical study conducted by researchers in Israel, Barda et al. In the analysis of over 800,000 vaccinated people, it was similarly revealed that COVID-19 vaccinations are associated with an elevated risk of myocarditis, about 1-5 cases per 100,000 vaccinations (4). The mechanism of this adverse effect has been ascribed to immune induced reactions mounted by the body to the viral proteins in the vaccine, similar to that after the actual viral infection.

COVID-19 vaccinations have also been associated with menstrual disturbances. A recent two-phase survey study, by researchers Muhaidat et al, consisting of a large cohort of about 2,200 participants, found that increased incidence of menstrual abnormalities, compared to pre-vaccination status. However, in nearly 94% of patients these abnormalities resolved within 2 months, indicating a relatively short duration of disturbances post-vaccine (7). Vaccine hesitancy is the refusal of vaccines despite their availability often due to anxiousness and nervousness (15). Fear of side effects and interaction with existing disease is a leading cause of vaccine hesitancy. A study of 6,500 cancer patients by the Leukemia Lymphoma Society found that fear of worsening their underlying cancer and lack of specific vaccine safety data for their disease was the most common cause of vaccine hesitancy (8% prevalence) in cancer patients (16).

Our study found that 41.2% of the sample population PD patients felt some worsening of their PD symptoms after COVID-19 vaccination. The *p* value of 0.0001 is not only highly statistically significant being < 0.05, but also clinically significant due to the large aforementioned relative risk of 1.65 and the odds ratio of 23:1. However, this worsening was mild in the majority of the cases (with 29 of 34 patients reporting a Likert score less than or equal to 2) and worsening commonly lasting between 24-48 hours. This finding is in line with several individual reports of temporary flare-ups of PD symptoms reported by several patients (5). Spontaneous worsening of baseline PD symptoms prior to vaccination was reported by only 1 of the 34 patients and was not considered to be statistically significant (*p*>0.05), conforming to current knowledge that spontaneous flare-ups without an inciting factor are uncommon in Parkinson’s Disease (6).

As depicted in **Table 7**, there was no statistically significant correlation between COVID-19 contraction and vaccine-induced flare-ups of PD symptoms (*p*=0.25). This implies that COVID-19 contraction induced antibodies are likely not responsible for causing PD worsening in vaccinated patients. This was important to look into since antibodies have been proven to cause brain, spinal cord, and nerve swelling (autoimmune encephalomyelitis) after vaccinations such as MMR and Influenza (17). Krammer et al. found that vaccination in individuals who contract COVID-19 induces a more robust immune response and higher titer of antibodies (18). No significant difference was noted in the debilitating disease rate between the vaccinated and unvaccinated elderly PD patients. Mortality was zero in both the groups. Curiously, there was also a mild to moderate, but statistically significant, correlation between vaccine hesitancy and PD symptom worsening (*r*=0.33). Vaccine hesitancy has been shown in numerous studies to be associated with increased anxiety after vaccination. Anxiety and stress are known to increase motor symptoms of PD (6). Therefore, there is a possibility that PD worsening was, in part, due to the noted occurrence of vaccine hesitancy among PD patients.

There was a mild but statistically significant positive correlation between severity of the vaccine’s general side effects (like fever, chills, pain) and PD worsening (*r*=0.28, *p*=0.04). This finding is important since it could suggest that the PD worsening was possibly related to a mechanism of generalized ‘non-specific immune reaction in the body’, simulating the body’s reaction to a mild infection. This reasoning is supported by Zheng et al. who found that the most common cause of worsening PD symptoms was any concurrent infection (6). Possible mechanisms include altered dopamine metabolism (altered presynaptic reuptake of L-DOPA and dopamine, respectively, and impaired packaging of neurotransmitters into vesicles), insensitivity to dopamine at receptor level, enhancement of ongoing neuroinflammatory processes in PD, or altered drug intake (19). In their study, Zheng et al. also found that tremor, gait, and dyskinesia were the most common symptoms to deteriorate, in that order. This is nearly in line with this study in which it was found that tremors, fatigue, and gait were the most exacerbated symptoms, in that order.

## Conclusion

Although there seems to be strong evidence of worsening of PD symptoms post COVID-19 vaccination, it is mostly mild and limited to a couple of days. Among the statistically significant positive correlations found with post vaccine PD worsening, the two that can provide a possible causative mechanism using existing scientific knowledge are vaccine hesitancy, likely via stress and anxiety and the extent of post-vaccine general side effects like fever, chills, pain, likely via simulating a mild systemic infection. The results of this study can spur PD patients who are hesitant to receive the vaccine to consider more strongly receiving the COVID-19 vaccine since there seem to be no detrimental, long-term effects on health. Those who were hesitant due to their underlying PD can perhaps make better informed decisions. Pre-existing research signifies that the effects of COVID-19, especially on elder populations who are unvaccinated, can be deadly (20). Therefore, this study can inform PD patients and encourage them to consider the benefits of getting vaccinated over the risk of contracting COVID-19.

The sample size of this research was quite small compared to typical clinical studies. Statistical correlation between PD worsening and Covid vaccination may not imply causation. Additional studies with larger sample sizes will be needed for further establishing an unequivocal relationship between PD symptoms and vaccination. The possible causative mechanisms proposed had correlations in the range of 0.28 and 0.33, which are considered weak to moderately strong correlations respectively and usually call for larger studies for further validation. Another limitation of the study is the confounding effect of subclinical infection. Subclinical SARS-CoV-2 infection can occur naturally in as high as 60% of cases. Finally, like a majority of Covid clinical studies, our study also used patients’ perception and recall ability of their symptoms. The study did not include any clinical or laboratory tests.

### Areas for future research

This study’s findings are catalysts to new areas of research. As noted, early onset PD patients (less than 50 years of age) represent a small percentage of all PD patients and manifest atypical symptoms (5). Therefore, this pool of patients is better suited to be studied as a separate group and future research can investigate this group and COVID-19 vaccines. When this study was designed, booster doses had not been introduced. Future research can also be expanded to include these COVID-19 booster doses.

## Data availability statement

The original contributions presented in the study are included in the article/**Supplementary Material**. Further inquiries can be directed to the corresponding author.

## Ethics statement

The studies involving human participants were reviewed and approved by University of Florida, Gainesville, and Shands Hospital IRB. The patients/participants provided their written informed consent to participate in this study.

## Author contributions

RS: primary investigator and data collection. OD: supervision and manuscript review. AA: data collection and manuscript review. VV-M: manuscript preparation and data collection. All authors contributed to the article and approved the submitted version.
