# Supplementary material for: Analyzing the effect of the COVID-19 vaccine on Parkinson’s disease symptoms
Source: Front Immunol. 2023 Jun 5;14:1158364. doi: 10.3389/fimmu.2023.1158364 (PMC10278957; doi:10.3389/fimmu.2023.1158364)

**Appendix A:** *University IRB Approval*

*Note:* Identifying information has been crossed out for privacy reasons.

*
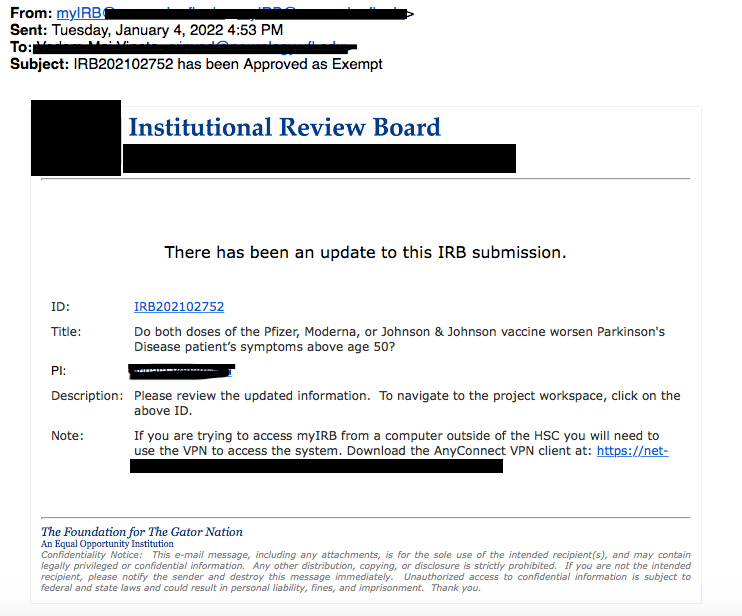
*

**Appendix B:** *Questionnaire Given to Patients at the Institute*

Note: You are invited to participate in a research project about “Effect of COVID-19 vaccination on Parkinson's Disease symptoms”. This survey should take about [10 to 15] minutes to complete. Participation is voluntary, and responses will be kept anonymous. You have the option to not respond to any questions that you choose. Participation or nonparticipation will not impact your relationship with the [Institute Name]. If you have any questions about the research, please contact:

Researcher: ______________ Email:_____________

Teacher: ________________ Email:____________

| 1. Sex | Male  Female  Non-Binary/Third Gender  Prefer not to say |
| --- | --- |
| 1. Race | White  Black or African American  American Indian or Alaskan Native  Asian  Native Hawaiian or Pacific Islander  Other |
| 1. Age Range | 50-59  60-70  70+ |
| 1. Please fill in the blank. I have had PD for _____ years. | Open Ended |
| 1. Have you contracted COVID during this pandemic? | Yes  No  Unsure |
| 1. On a scale of 0-5 how hesitant were you to receive the COVID-19 vaccine | Sliding Scale from 0-5 |
| 1. If you were hesitant, was it due to your PD? | Yes  No  Unsure |
| 1. Have you received both doses of the COVID-19 vaccine? | Yes  No  Only 1  I received J&J, so only 1 |
| 1. Which COVID-19 vaccine did you receive? | Moderna  Pfizer  J&J  None |
| 1. Which of the following side effects did you have from the COVID-19 vaccine? (Check all that apply) | Check all that apply; list of 8 common COVID-19 vaccination side effects (including fatigue, swelling.) |
| 1. On a scale of 0-5, how severe were the COVID-19 vaccine side effects? | Sliding Scale from 0-5 |
| 1. How long did the COVID-19 vaccine side effects last? | <24 hours  28-48 hours  >48 hours |
| 1. Which of the following PD symptoms are the most common/troublesome to you? | Check all that apply; list of 11 common PD symptoms |
| 1. On a scale of 0-5, how much do the symptoms you selected above interfere with daily activities | Sliding Scale from 0-5 |
| 1. Were you able to continue your daily activities, chores, and/or exercise regularly after getting vaccinated? | Yes  No |
| 1. On a scale of 0-5 how would you rate the extent to which your PD symptoms worsened after getting the COViD-19 vaccine? | Sliding Scale from 0-5 |
| 1. Which symptoms were most affected and in what way? | Check all that apply |
| 1. How long did worsening last? | <24 hours after vaccination  24-48 hours after vaccination  >48 hours after vaccination  *If you chose >48 hours please specify:: _____ after vaccination* |
| 1. Did it occur after each COVID-19 vaccine dose? | Yes  No |
| 1. Which of the following PD medications are you taking? | Check all that apply; list of 26 common PD medications |
| 1. Did you change your medications or your medication schedule days leading up to (or after) getting vaccinated? | Yes  No |
| 1. Have your PD symptoms spontaneously (in absence of infection, hospitalization, stress, and/or discontinuation of medication) worsened in the past 12 months? | Yes  No |

**Appendix C:** *Informed Consent*

**Title of Project:** Effect of the COVID-19 Vaccination on Parkinson’s Disease Symptoms.

**Principal Investigator:** [Name]

1. **Purpose of the Study:** The purpose of this study is to determine whether COVID-19 vaccines affect PD patients and their symptoms.
2. **Procedures to be Followed:** You will be asked to answer 22 questions on a survey.
3. **Duration:** It will take about 10-15 minutes to complete the survey.
4. **Research Benefits:** There is no direct benefit to you for being in this study. There may be a benefit to others depending on the results of this study
5. **Research Risks:** Your participation in this study involves no physical risks. As noted before, you have the ability to opt out of any particular question with no penalty. There is a risk that information received by these authorized persons or agencies could be passed on to others beyond your authorization and not covered by HIPAA.
6. **Statement of Confidentiality:** Your participation in this research is confidential. The survey does not ask for any information that would identify who the responses belong to. In the event of any publication or presentation resulting from the research, no personally identifiable information will be shared because your name is in no way linked to your responses.
7. **Right to Ask Questions:** Please contact _____________ at _________ with questions or concerns about this study
8. **Payment for Participation:** Participants will receive no payment for participating in this study.
9. **Privacy Authorization:** [University Name] will be allowed to collect, use, and/or give out your medical information, but only to:
10. Other researchers whose research is approved by an Institutional Review Board (IRB)
11. The sponsor of a study (like a drug company), the Food and Drug Administration, the Department of Health and Human Services, the Office of Human Research Protections or other Government agencies

**10. Voluntary Participation:** Your decision to be in this research is voluntary. You can't stop at any time. You do not have to answer any questions you do not want to answer. If you do not want to take part you can call _____ at any time to let me know. If you choose not to take part, this will have no effect on your current or future healthcare at [Institute Name] at the [University Name]. If you have any questions about your rights as a research subject, you can phone the IRB at ______.

Completion and return of this survey implies that you have read the information in this form and consent to take part in this research.

**Appendix D:** *Survey Responses*


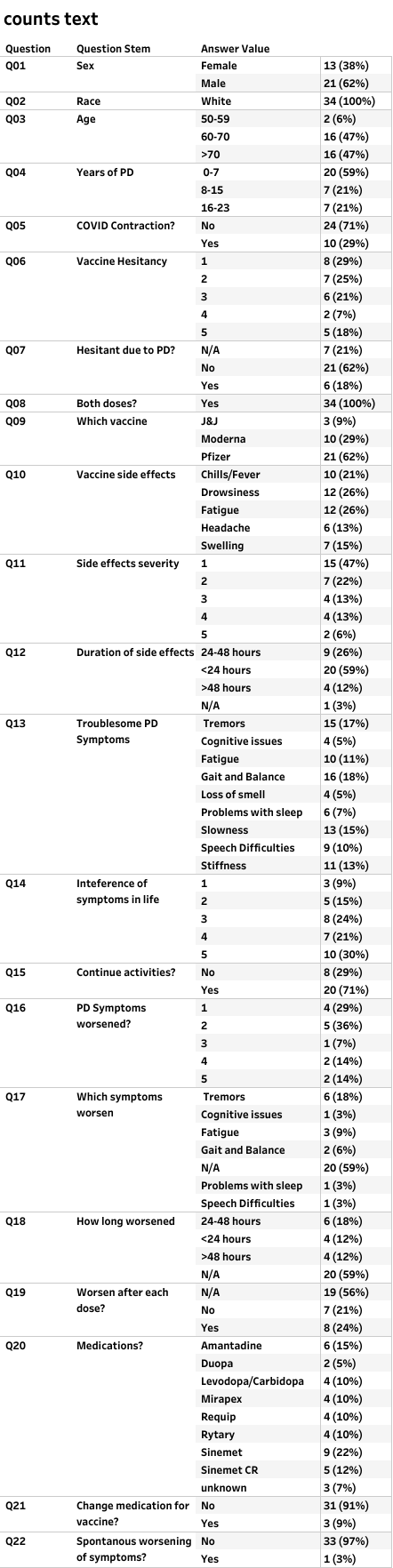

Supplement: Supplementary file 1 [file DataSheet_1.docx]
